# Case Report: Phosphaturic mesenchymal tumor presenting solely as knee pain without hypophosphatemia

**DOI:** 10.3389/fonc.2025.1597194

**Published:** 2025-05-08

**Authors:** Qianqian Cao, Cheng Cheng, Zhipeng Dai

**Affiliations:** Zhengzhou University People’s Hospital, Henan Provincial People’s Hospital, Zhengzhou, Henan, China

**Keywords:** phosphaturic mesenchymal tumors, tumor-induced osteomalacia, fibroblast growth factor 23, pain, hypophosphatemia, osteomalacia

## Abstract

Phosphaturic mesenchymal tumor (PMT) is a rare tumor disease that often leads to tumor-induced osteomalacia (TIO). The typical manifestations of this disease include hypophosphatemia and osteomalacia. The main symptom in most PMT patients is diffuse pain throughout the body. However, we report a PMT patient with typical histological features but without hypophosphatemia and TIO. This patient only presented with pain in the right knee joint. At 6 months and 1 year after surgery, follow-up revealed that the patient’s swelling and pain in the right knee joint had disappeared. Moreover, the imaging and biochemical examinations conducted at the local hospital showed normal results.

## 1 Introduction

Phosphaturic mesenchymal tumor (PMT) is a rare tumor originating from bone and soft tissue, usually presenting as tumor-induced osteomalacia (TIO) (1). TIO is a paraneoplastic syndrome that can be caused by various other diseases in addition to PMT, such as odontogenic fibroma, and hemangiopericytoma (2). PMT can produce a variety of phosphatonins, including fibroblast growth factor 23 (FGF-23), secreted frizzled-related protein 4 (sFRP-4), and matrix extracellular phosphor-glycoprotein (MEPE) (3, 4). Among these phosphatonins, FGF-23 plays the most critical role. FGF-23 can act on the epithelial cells of the proximal renal tubules, reducing the reabsorption of phosphate while promoting its excretion (5). This process leads to the continuous consumption of phosphate in the body, ultimately resulting in hypophosphatemia in patients. In 1987, the concept of PMT was proposed by Weidner and Santa (6). PMT was included in the classification of tumors of soft tissue and bone in 2013 (7). The non-phosphaturic variant of PMT is uncommon (8–10). So far, only slightly more than 1,000 cases of TIO have been documented worldwide, including those caused by PMT (11). Studies have shown that PMT is the main type of tumor that causes TIO (12). The clinical manifestations of TIO are mainly related to tumor-induced hypophosphatemia, rather than the direct effect of the tumor itself (13). Since hypophosphatemia can be caused by a variety of different etiologies, this makes the clinical manifestations of TIO non-specific. Patients often present with a variety of symptoms such as fatigue, bone pain, fractures, and muscle weakness (14). Because of its rare occurrence, relatively small size, slow benign growth, and nonspecific systemic manifestations, it typically takes 5–7 years from tumor appearance to disease diagnosis (15). This case report discusses a rare case of PMT of the proximal right fibula, and the patient was free of hypophosphataemia and osteomalacia. It is hoped that this case will provide a valuable clinical reference for the early diagnosis and treatment of this condition.

## 2 Case presentation

A 65-year-old female patient presented with persistent pain and discomfort in the right knee for 3 months with no obvious trigger, and the pain worsened with activity. After taking anti-inflammatory and analgesic drugs at home, the patient’s knee pain symptoms were not significantly relieved, so she went to Henan Provincial People’s Hospital. The patient had no muscle or bone pain in other parts of the body except for the right knee. Physical examination of the patient revealed a slight swelling of the right knee joint, normal skin temperature around the knee joint, pronounced pressure pain at the posterolateral side of the knee joint, and normal muscle strength and tone. She had no past medical history other than a history of cerebral infarction and hypertension, and no family members were found to have a history of similar illnesses.

### 2.1 Diagnostic assessment

**Figure 1:**
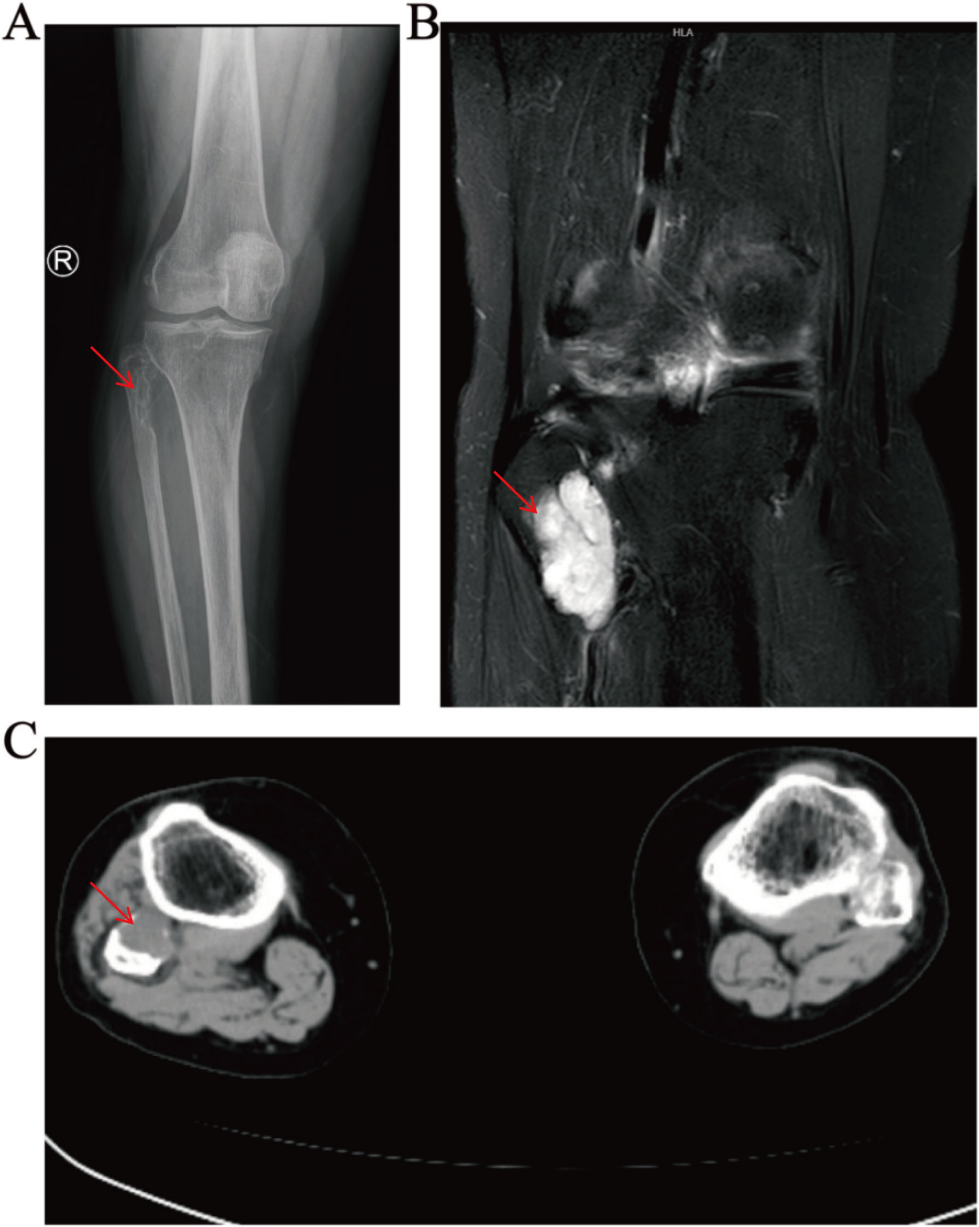
Preoperative imaging of the patient. **(A)** X-ray: bone defect in the proximal part of the fibula with uneven bone density, **(B)** enhanced MRI: tibiofibular interosseous soft-tissue mass, **(C)** CT: fibular head and tibiofibular interosseous soft-tissue mass.

**Figure 2:**
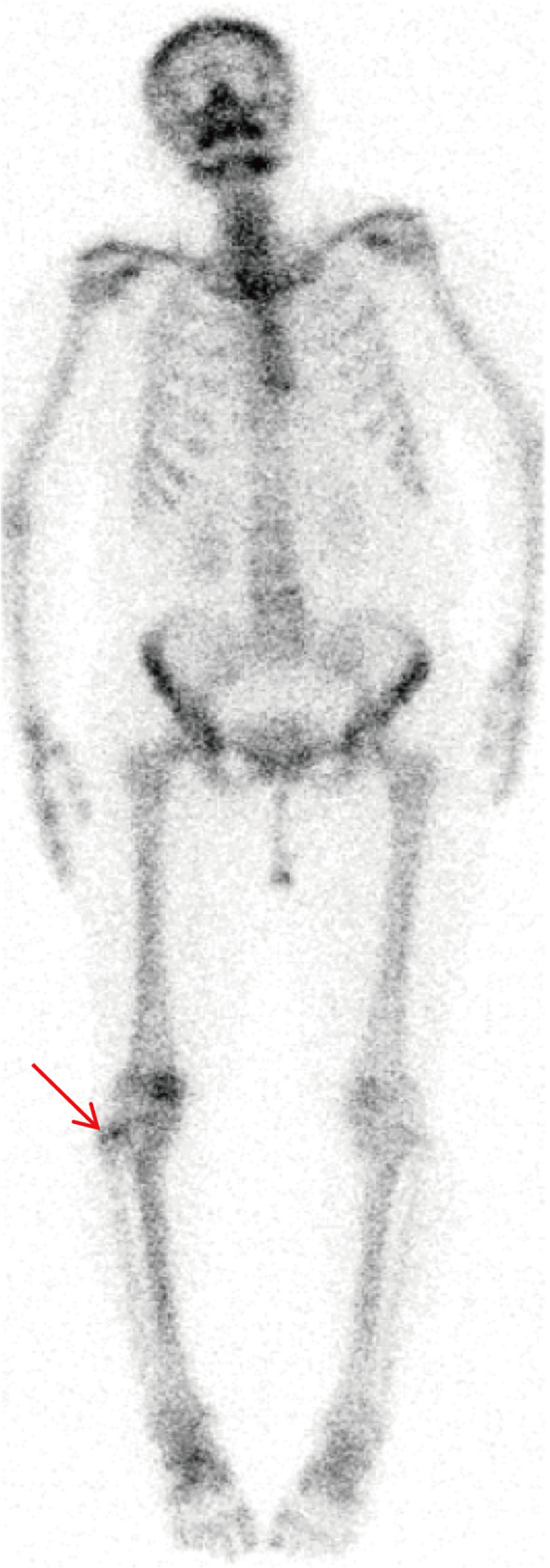
99mTc-MDP scan showing a dense shadow of radioactivity distribution in the right proximal fibula.

The above signs and symptoms are of limited value in the diagnosis of the disease. We then performed further laboratory and imaging studies on the patient. It is worth noting that the patient’s blood routine test results are normal, and so is the serum phosphorus level (**Table 1**). Additionally, the anteroposterior X-ray of the patient’s right knee joint showed a bone defect in the proximal end of the right fibula, with heterogeneous bone density. (**Figure 1A**). Integrating the aforementioned findings, we hypothesized that the patient might have a bone tumour. Therefore, the patient underwent a series of in-depth examinations, including tumour marker laboratory tests, whole-body bone imaging, computed tomography (CT), and magnetic resonance imaging (MRI). As shown in **Supplementary Table S1**, the tumor marker tests, including AFP, CEA, CA242, CA125, CA153, CA199, TPSA, FPSA, β-HCG, HGH, FE, and NSE, were all within normal range. 99mTc-MDP whole-body bone imaging revealed a focal area of increased radiotracer uptake at the proximal end of the right fibula, while the remainder of the skeleton exhibited no significant abnormalities in radiological distribution (**Figure 2**). The CT results of the right knee joint showed a soft-tissue mass at the proximal part of the right fibula (**Figure 1C**). Additionally, the results of plain and enhanced MRI of the right knee joint indicated a soft-tissue mass at the proximal part of the right fibula, with bone resorption of the proximal fibula, suggesting a neoplastic lesion (**Figure 1B**). To identify the patient’s tumor type and formulate the subsequent treatment plan, we conducted a needle biopsy on the patient’s tumor tissue. The biopsy results indicate that it is suspected to be PMT. In light of the characteristics of the PMT’s mechanism of action, we measured the 24-hour urinary electrolyte levels of the patients prior to surgery. The results indicated that the patients’ 24-hour urinary phosphorus levels decreased, while the 24-hour urinary calcium, chlorine and sodium levels increased (**Table 2**).

**Table 1:** Post-admission and postoperative serum electrolyte test results.

Parameter	Post-admission Value	Postoperative Value	Normal range
K	4.11	4.51	3.5-5.3mmol/L
Na	141	140	137-147mmol/L
CL	105	107	99-110mmol/L
Ca	2.33	2.42	2.11-2.52mmol/L
P	1.19	1.34	0.85-1.51mmol/L

K, Potassium; Na, Sodium; CL, Chlorine; Ca, Calcium; P, Phosphorus.

**Table 2:** Results of 24-hour preoperative urine electrolyte tests.

Parameter	Value	Normal range
24hK	51.52	25-100mmol/24h
24hNa	384↑	130-136mmol/24h
24hCL	352↑	170-250mmol/24h
24hCa	17.98↑	2.5-7.5mmol/24h
24hP	19.10↓	23-48mmol/24h

K, Potassium; Na, Sodium; CL, Chlorine; Ca, Calcium; P, Phosphorus.

### 2.2 Treatment

**Figure 3:**
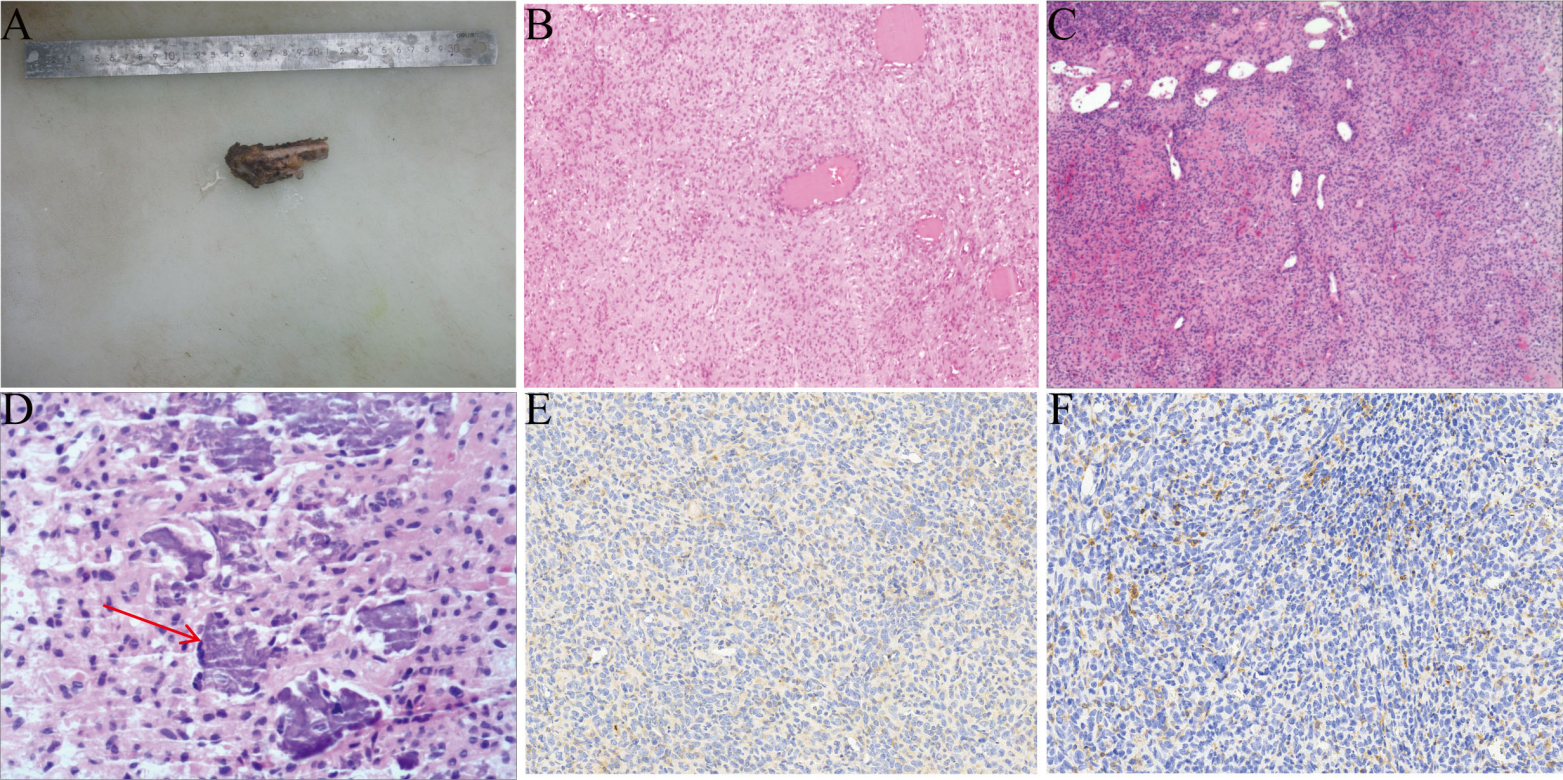
The pathology and immunohistochemistry of this case. **(A)** This is the tumor removed through surgery. **(B-C)** The tumor is rich in blood vessels. **(D)** Focal flocculent calcifications. **(E, F)** The expression of CD56 and SATB2 (**×**20).

Previous research findings indicate that surgical resection of PMT represents the curative treatment approach (16). Therefore, we decided to surgically remove the diseased part of the proximal fibula and send the excised sample for pathological examination (**Figure 3A**). The tumor in this patient has distinct histological features: the tumor tissue is highly vascularized, and there are focal flocculent calcifications. (**Figures 3B, C**). The immunohistochemical results show: Vimentin (+), SMA (+), Bcl-2 (+), CD56 (+), CD34 (+), CD31 (+), Ki67 (1%+), ERG (+), CK (AE1/AE3) (-), INI-1 (+), SATB2 (+), SSTR2 (-) (**Figures 3E, F**). Based on the histological features and immunohistochemical results, the final diagnosis is PMT.

### 2.3 Outcome and follow-up

After the surgery, the patient’s right knee joint pain was significantly alleviated. The results of blood routine tests, liver and kidney function tests, and serum electrolyte tests one week after the surgery were all normal (**Table 1**). At six months and one year after the surgery, we followed up with the patient separately. The patient’s pain and swelling in the right knee joint had disappeared, and the biochemical and imaging examination results at the local hospital were all normal, with no signs of recurrence.

## 3 Discussion

### 3.1 Clinical features

PMT is an exceptionally rare neoplasm, typically observed in middle-aged individuals and infrequently encountered in elderly patients (17–20). It has the potential to arise in any site within the skeletal system, albeit with a predilection for the appendicular skeleton, skull, and paranasal sinuses (21–24). PMTs are usually less than 5 cm in size and rarely cause localized pain related to the tumor site (4). The typical clinical manifestation of PMT is diffuse musculoskeletal pain, which is caused by secondary osteomalacia due to phosphate loss in patients (25, 26). This leads to an average time from symptom onset to final diagnosis that is typically as long as three years (27, 28). Notably, our patient exhibited distinct clinical characteristics. The patient in question was a 65-year-old female who initially complained of right knee pain lasting for 3 months, without any concurrent musculoskeletal pain upon presentation. The clinical presentation of this patient deviated from the conventional paradigm of PMT, highlighting the importance of considering individual variability in clinical diagnosis and expanding our diagnostic approach to facilitate earlier recognition of this uncommon neoplasm.

### 3.2 Pathogenesis

Most PMT-associated TIO is attributed to the overexpression of FGF-23 (29). FGF-23 diminishes the capacity of proximal renal tubules to reabsorb phosphate and augments phosphate excretion by the kidneys (30, 31). Furthermore, FGF-23 inhibits the activity of 1-α-hydroxylase, subsequently decreasing the synthesis of 1,25-dihydroxycholecalciferol (4). Other phosphatonins secreted by PMT, such as fibroblast growth factor 7 (FGF-7), secreted frizzled-related protein 4 (sFRP-4), and matrix extracellular phosphoglycoprotein (MEPE), can also lead to abnormal phosphate metabolism (32). Ultimately, these alterations result in hypophosphatemia and osteomalacia. Nonetheless, our patient exhibited neither hypophosphatemia nor any clinical manifestations of osteomalacia. Likewise, Folpe et al. identified cases lacking a known history of phosphaturia (17). They speculated that, in such instances, the tumor may secrete inactive or insufficient FGF-23, or possibly none at all. An alternative explanation posits that the patient possesses the capacity to compensate for increased FGF-23 secretion via alternative mechanisms. This may encompass compensatory regulation by other pathways or hormones involved in phosphate metabolism, thereby preventing the onset of hypophosphatemia and osteomalacia. Since we did not test for FGF-23, we are unable to speculate whether there is a compensatory mechanism in our patient. We speculate that the absence of hypophosphatemia and osteomalacia in our patient may be attributed to the early detection and resection of the tumor before the substantial production of humoral factors.

### 3.3 Imaging features

On CT examination, bone lesions in PMT patients typically present as osteolytic, and often contain internal matrix (33). On MRI examination, PMT usually shows isointensity on T1, hyperintensity on T2, with marked enhancement, and often accompanied by areas of low T2 signal (34). In addition, since PMT is a metabolically active tumor, radionuclide scans based on somatostatin receptor expression are of particular importance. In clinical practice, 99mTc-sestamibi scintigraphy, 111In-pentetreotide scintigraphy, 68Ga-DOTATATE PET/CT, and 18F-FDG PET/CT are commonly used to identify PMT (35). Existing research has shown that 68Ga-DOTATATE imaging is particularly sensitive in detecting occult tumors (36). CT examination of the patient revealed a cystic hypodense lesion with localized osteolytic alterations in the proximal region of the right fibula. MRI examination further elucidated that the proximal aspect of the right fibula exhibited isosignal intensity on T1-weighted images and high signal intensity on T2-weighted images. During contrast-enhanced scanning, the lesion area demonstrated significant heterogeneous enhancement. Furthermore, whole-body bone scintigraphy indicated the presence of a focal area of increased radiolabeled tracer uptake in the proximal portion of the right fibula. The imaging features of the patient are highly consistent with the typical imaging characteristics of PMT reported previously.

### 3.4 Pathologic and immunohistochemical features

PMT is a rare bone tumor with unique pathological features. There are four subtypes, namely osteoblastoma-like type, non-ossifying fibroma-like type, ossifying fibroma-like type, and mixed connective tissue type (6). Among these subtypes, the mixed connective tissue type is the relatively most common variant (17). The histological features of PMT include a small to moderate amount of bland spindle cells, small cell nuclei, inconspicuous nucleoli, a small number of mitotic figures, dirty calcified stroma, hemangiopericytoma-like vessels, osteoclast-like giant cells, fat, microcysts, hemorrhage, and other pathological changes (37, 38). Although immunohistochemical examination is not absolutely necessary for the diagnosis of PMT, it still has certain value in auxiliary diagnosis. Relevant studies have shown that in PMT cases without TIO, FGF-23 immunostaining is usually negative (39). In addition, PMT tumor cells can express a variety of markers, including vimentin, D2-40, SSTR2, ERG, FLI1, CD56, SMA, and SATB2 (10, 40–43). The research by Houang et al. pointed out that FGF-23 and SSTR2 are relatively sensitive for the diagnosis of PMT, but their specificity is relatively low (41). The FN1-FGFR1 fusion gene can be detected in most PMT cases, while the FN1-FGF1 fusion gene can be detected in a few cases (44, 45). Previous studies have shown that immunohistochemical detection of FGFR1 is helpful for the diagnosis of PMT, and meanwhile, RT-PCR detection of FGF-23 is a relatively sensitive method for diagnosing PMT (46). The tumor tissue in our patient exhibited typical histological features of PMT, including abundant calcified stroma and rich vasculature. Immunohistochemical results showed that the tumor cells expressed Vimentin, SMA, Bcl-2, CD56, CD34, CD31, Ki-67, ERG, RB, INI-1, and SATB2, but were negative for SSTR2 and CK. The detection of FGF-23 expression in tumor tissues is of significant value for differentiating PMT from other mesenchymal tumors with overlapping histological features (9). However, due to the retrospective nature of the case report, the expression of FGF-23 was not detected, which is a limitation of this study. These findings are highly consistent with the histological features and immunophenotype of PMT reported in previous studies. Ultimately, these characteristics enabled us to accurately diagnose the patient with PMT. Although the biopsy initially suggested that the lesion in this case was PMT, it is generally not recommended to perform biopsies on patients suspected of having PMT. This is because the biopsy procedure carries certain risks, such as the potential for tumor cell implantation, which may lead to local recurrence (47). This, in turn, can adversely affect disease control and subsequent treatment for the patient.

### 3.5 Treatments

In most cases, PMT is benign and can be completely cured by surgical excision. Negative surgical margins are a key factor in reducing the risk of recurrence (48). Radiotherapy is applicable for PMT that cannot be surgically resected (4). For cases where the tumor cannot be clinically localized, a pharmacological treatment approach, including the administration of phosphorus and calcitriol, is recommended (7, 37). Our patient resumed a normal diet after surgery and did not receive pharmacological treatment. The patient’s knee pain significantly improved after the surgery. We followed up with the patient at 6 months and 1 year postoperatively. Biochemical and imaging tests performed at the local hospital showed no signs of recurrence.

## 4 Conclusion

In this case, we present a PMT patient without hypophosphatemia and osteomalacia. The patient only presented with pain in the right knee joint. After a pathological examination of the tumor tissue, we found typical histological features of PMT. Therefore, when dealing with non-phosphaturic variant of PMT patients, histopathological examination of the tumor tissue is mandatory for accurate diagnosis and also for formulating subsequent treatment plans.

## Data Availability

The original contributions presented in the study are included in the article/supplementary material. Further inquiries can be directed to the corresponding author.
