# Supplementary material for: Case Report: Phosphaturic mesenchymal tumor presenting solely as knee pain without hypophosphatemia
Source: Front Oncol. 2025 May 8;15:1597194. doi: 10.3389/fonc.2025.1597194 (PMC12094952; doi:10.3389/fonc.2025.1597194)
Supplement: Supplementary file 1 [file Table1.docx]

**TableS1** **Results of tumor marker tests**

| **Parameter** | **Value** | **Normal range** |
| --- | --- | --- |
| AFP | 7.12 | < 20 ng/ml |
| CEA | 2.4 | < 5 ng/mL |
| CA-242 | 4.39 | < 20 KU/L |
| CA-125 | 13.56 | < 35 u/ml |
| CA-153 | 18.28 | < 35 u/ml |
| CA-199 | 10.9 | < 35 KU/L |
| T-PSA | 1.59 | < 5 ng/mL |
| F-PSA | 0.18 | < 1 ng/ml |
| B-HCG | 0.52 | < 3 mIU/mL |
| HGH | 0.83 | < 7.5 ng/ml |
| FE | 77.57 | < 219 ng/mL |
| NSE | 3.87 | < 13 ng/ml |

AFP, Alpha-Fetoprotein; CEA, Carcinoembryonic Antigen; CA, Carbohydrate Antigen; TPSA, Total Prostate Specific Antigen; F-PSA, Free Prostate Specific Antigen; B-HCG, β-human Chorionic Gonadotropin; HGH, Human Growth Hormone; FE, Ferritin; NSE, Neuron Specific Enolase.
